# Association Between State-Issued COVID-19 Vaccine Mandates and Vaccine Administration Rates in 12 US States and the District of Columbia

**DOI:** 10.1001/jamahealthforum.2022.3810

**Published:** 2022-10-28

**Authors:** Mara Howard-Williams, Rieza H. Soelaeman, Leah S. Fischer, Russell McCord, Robin Davison, Christopher Dunphy

**Affiliations:** 1 COVID-19 Response Team, Centers for Disease Control and Prevention, Atlanta, Georgia; 2 National Center for Injury Prevention and Control, Centers for Disease Control and Prevention, Atlanta, Georgia

## Abstract

**Importance:**

Some US states have issued COVID-19 vaccine mandates; however, the association of these mandates with vaccination rates remains unknown.

**Objective:**

To examine the association between announcing state-issued COVID-19 vaccine mandates that did not provide a test-out option for workers and the vaccine administration rates in terms of state-level first-dose vaccine administration and series completion coverage.

**Design, Setting, and Participants:**

This cross-sectional study used publicly available, state-level aggregated panel data to fit linear regression models with 2-way fixed effects (state and time) estimating vaccine coverage changes 8 weeks before and 8 weeks after a state-issued COVID-19 vaccine mandate was announced. Mandates were announced on or after July 26, 2021, and were included only if they went into effect before December 31, 2021. Data were included from 13 state-level jurisdictions with a vaccine mandate in effect as of December 31, 2021, that did not allow recurring testing in lieu of vaccination (mandate group), and 14 state-level jurisdictions that allowed a test-out option and/or did not restrict vaccine requirements (comparison group).

**Interventions/Exposures:**

The event of interest was the announcement of a state-issued COVID-19 vaccine mandate applicable to specific groups of workers.

**Main Outcomes and Measures:**

The outcome measures were state-level daily COVID-19 vaccine first-dose administration and series completion coverage, reported as mean percentage point changes.

**Results:**

Of 5 508 539 first-dose administrations in the 8-week postannouncement period, an estimated 634 831 (11.5%) were associated with the mandate announcement. First-dose administration coverage among 13 jurisdictions increased starting at 3 weeks after the mandate announcement, with statistically significant differences of 0.20, 0.33, 0.39, 0.45, 0.49, and 0.59 percentage points higher than the referent category coverage of 62.9%. Increases in vaccine series completion coverage were observed from 5 to 8 weeks after the announcement, but statistically significant differences from the referent category coverage of 56.3% were observed only during weeks 7 and 8 after the announcement (both differed by 0.2 percentage points; *P* = .05 and *P* = .02, respectively).

**Conclusions and Relevance:**

The findings of this cross-sectional event study suggest that the announcement of state-issued vaccine mandates may be associated with short-term increases in vaccine uptake. This observed association may be a product of both a direct outcome experienced by groups governed by the mandate as well as the spillover outcome due to a government signaling the importance of vaccination to the general population of the state.

## Key Points

**Question:**

What is the association between the announcement of state-issued COVID-19 vaccine mandates for workers in the US and vaccine administration rates in terms of first-dose administration and series completion coverage?

**Findings:**

In this cross-sectional study of 12 states and the District of Columbia with state-issued COVID-19 vaccine mandates, the state-issued vaccine mandates were associated with an increase in the percentage of the population receiving the first dose of the vaccine compared with 14 states without such mandates.

**Meaning:**

The findings of this study suggest that state-issued COVID-19 vaccine mandates may have encouraged state populations to seek vaccinations, even if the population was not specifically required to do so under the order.

## Teaser

This cross-sectional study examines the association between the announcement of state-issued COVID-19 vaccine mandates for workers and the rates of vaccine administration in terms of first-dose vaccine administration and series completion coverage, in 12 US states and the District of Columbia from May 31 to October 12, 2021.

## Introduction

State and local jurisdictions in the United States have effectively used mandatory vaccination laws to control and prevent transmission of infectious diseases in various settings since the early 19th century, notably ending the smallpox epidemic.^1^ Studies have examined the outcomes of vaccination laws and recommendations in circumscribed populations, such as school-age children and health care workers, for whom mandates are historically engrained. For example, the public health benefits accrued from vaccine mandates for daycare and school entry are well established.^2,3,4,5^ Immunization mandates, found to be more effective than recommendations, have been associated with increased vaccination uptake prior to daycare or school entry.^6,7^

Mandatory vaccination laws are grounded in the US Constitution’s Tenth Amendment granting states the right to enact laws that promote the public health and safety of the state’s inhabitants, commonly referred to as “police power.” In 1809, Massachusetts passed a law allowing local governments to require vaccination, and in 1855, it became the first state to issue a mandate for smallpox vaccination applicable to the general population; this served as a catalyst for other states to implement laws for mandatory vaccinations.^8,9,10^ By the end of the 19th century, 13 states required vaccination for schoolchildren and 11 states had vaccine mandates for the rest of the states’ inhabitants.^11^ As of 2022, all 50 states require schoolchildren to receive multiple vaccines to attend public schools.^12^ The Supreme Court of the United States has repeatedly upheld the authority for states to mandate vaccination under the states’ police power, since 1905 as in *Jacobson v Massachusetts*^13^ and *Zucht v King*.^14^ Studies of mandates specific to health care workers have found vaccine mandates for influenza to be more effective than vaccine recommendations alone.^15,16,17,18,19^

In the context of COVID-19, some state governments began mandating vaccines applicable to certain groups of workers after the US Department of Justice’s Office of Legal Counsel publicly released a memorandum on July 26, 2021,^20^ stating that the emergency use authorization status of COVID-19 vaccines did not preclude a public or private employer’s ability to mandate that employees get vaccinated. States responded by issuing mandates specific to worker groups, including those in health care, long-term care facilities, schools, congregate facilities, and the government. States varied in the content of their mandates, including how many worker groups are required to be vaccinated, what exemptions are allowed, and whether workers could undergo recurring testing in lieu of vaccination (“test-out option”). As of February 2022, the Supreme Court declined to hear at least 3 challenges to states’ COVID-19 vaccine mandate cases, effectively leaving the vaccine mandates in place.^21,22,23^

By the end of December 2021, 24 state-level jurisdictions had announced a vaccine requirement that applied to at least 1 group of workers. However, relevant literature addressing the effectiveness of current state-issued COVID-19 vaccine mandates is still minimal.

This study examined the association between the announcement of state-issued COVID-19 vaccine mandates for workers and the vaccine uptake in terms of first-dose vaccine administration and series completion coverage. The results of this analysis could inform policy makers on the extent to which government mandates may have been associated with increases in vaccination rates and how these mandates may be used to mitigate the spread of infectious diseases during public health emergencies.

## Methods

This cross-sectional study was reviewed by the Centers for Disease Control and Prevention (CDC) and was conducted consistent with applicable federal law and CDC policies, including 45 Code of Federal Regulations (CFR) part 46, 21 CFR part 56; 42 US Code (USC) §241(d); 5 USC §552a; and 44 USC §3501 et seq.

### Data Sources

#### State-Issued COVID-19 Vaccine Mandates

Data on state-issued vaccine mandates were obtained from laws identified on state government websites. Laws were analyzed and coded to extract mitigation policy variables for vaccine mandates, including the date they were announced, the effective date, and specific information about the content of the orders, such as the groups of workers to whom they applied. State-issued vaccine mandates were defined as requirements for a group of workers to (1) be vaccinated with no test-out option except for those with approved medical or religious exemptions; or (2) be vaccinated and undergo recurring testing, regardless of exemption status. All coding underwent secondary review and quality assurance checks by 2 or more raters; on agreement among all raters, the coding and analyses were published in publicly available data sets.^24,25^

#### Inclusion Criteria

Data from the 50 states and the District of Columbia (collectively, “states”) were analyzed for inclusion. No federal, local, tribal, or territorial jurisdictions were included. States were included in the mandate group if they issued at least 1 mandate requiring full vaccination (to have completed a primary series from any of the 3 authorized vaccines [Ad26.COV2.S (Janssen/Johnson & Johnson), mRNA-1273 (Moderna), or BNT162b2 (Pfizer-BioNTech)]), with no test-out option in effect on or before December 31, 2021. States included in the comparison group either (1) issued a mandate that allowed a test-out option, or (2) did not issue any type of mandate and did not otherwise restrict mandates, such as by issuing mandate prohibitions. The analysis included data from 13 mandate group states (California, Colorado, Connecticut, the District of Columbia, Maine, Massachusetts, Nevada, New Mexico, New York, North Carolina, Oregon, Rhode Island, and Washington) and 14 comparison group states (Delaware, Hawaii, Illinois, Kentucky, Louisiana, Maryland, Minnesota, New Jersey, Ohio, Pennsylvania, Virginia, Vermont, West Virginia, and Wisconsin). States that issued mandate prohibitions (n = 22) were excluded from the comparison group because prohibitions are best characterized as an opposite intervention rather than as a control and were enacted months prior to the time frame for mandate announcements. The announcement date was defined as the first date a law was formally announced by the governing body, regardless of the type of mandate. The effective date was defined as the last possible date by which individuals must be fully vaccinated as defined by the CDC^26^ (2 weeks after the last dose of a primary series) to comply with the law. In states where effective dates were postponed, the latest effective date within the study period was used for analysis.

#### State-Level Vaccination and Epidemiological Data

State-level time series data on vaccine first-dose administration, vaccine series completion, the percentage of total state population fully vaccinated, and COVID-19 cases per 100 000 population were obtained from the CDC COVID Data Tracker public use data (dated June 6, 2022).^27,28,29^ Each line of observation in the daily time series data set contained variables on vaccination, epidemiology, and vaccine mandate status for a state on a given calendar date. First-dose administration is defined as the date an individual received the first dose of a 2-dose series or the only dose of a single-dose series. Vaccine series completion is defined as the date an individual received the second dose of a 2-dose series or the only dose of a single-dose series. The vaccine series completion date does not include the 2-week post-series completion time frame to be considered fully vaccinated under CDC’s definition.^30^

### Statistical Analysis

#### Regression Model

An event study design was used to examine 2 outcomes^31,32^: (1) the percentage of a state’s population with first doses of vaccination, and (2) the percentage with vaccine series completion. Changes in the percentage of the population with first-dose administration and vaccination series completion coverage for the mandate group states were modeled using linear regression, including 15 mutually exclusive weekly time variables relative to the date of the state-issued vaccine mandate announcement. Centering the time component around the event of interest allows comparison across states, as each state had a different vaccine mandate announcement date. The analysis was limited to the period of 8 weeks before and 8 weeks after the mandate announcement date. The time from the mandate announcement date was categorized into a total of sixteen 7-day periods, covering 8 weeks (50-56 days) before the mandate announcement to 8 weeks after the announcement. The referent category included observations from 0 to 2 weeks prior to the announcement date for mandate group states and included data points from the comparison group states. For mandate group states, data points were included from 8 weeks before and after the mandate announcement date for that state. For comparison group states, the data points were included from May 31, 2021 (8 weeks before the earliest mandate announcement on July 26), through October 12, 2021 (8 weeks after the most recent mandate announcement on August 17), to ensure that each mandate group state data point had corresponding comparison group state data points against which they could be compared. The variables the model controlled for were state and time (calendar date) 2-way fixed effects, previous-week 7-day rolling average new cases per 100 000 population, percentage of population fully vaccinated (for the first-dose model), and whether the observation date occurred before or after the state’s mandate effective date. Observations occurring on the mandate announcement date were excluded from the analyses. Percentage point changes in coverage relative to the referent category were computed from the model coefficients for both outcomes and graphed. The coefficient on the weekly time variable can be interpreted as the average percentage point difference, attributed to the mandates, in coverage for that week compared with the referent category. For all regression coefficients, a 2-tailed *t* test *P* value of less than or equal to 0.05 was considered statistically significant. Seven-day rolling averages of new cases per 100 000 population were calculated using the user-written Stata package -*asrol*-. All analyses were performed in Stata SE, version 17 (StataCorp LLC).

#### Additional Vaccinations Associated With Mandate Announcement

To illustrate the tangible application of the model estimates, we estimated the number of vaccinations that would have occurred in the absence of vaccine mandates (the counterfactual scenario). The additional vaccinations potentially attributable to the announcement of the mandate over the 8 weeks after the mandate announcement were calculated using model estimates by taking the difference between the actual (observed) cumulative doses at the end of the week and the expected cumulative doses in the counterfactual scenario.

## Results

Of the 24 state-level jurisdictions that had announced a vaccine requirement that applied to at least 1 group of workers by the end of December 2021, 13 met the inclusion criteria for the mandate group states. The final analytic data set contained 3346 state-day observations, with 13 mandate group states (Table) contributing 1456 data points and the 14 comparison group states contributing 1890 data points. Of the 13 mandate group states, 11 states had mandates applicable to health care workers, 11 had mandates applicable to government workers, 8 had mandates applicable to long-term care facility workers, 7 had mandates applicable to K-12 school workers, and 4 had mandates applicable to congregate facility workers. One state (California) had mandates applicable to all 5 groups, 6 states had mandates applicable to 4 groups, 2 states had mandates applicable to 3 groups, 3 states had mandates applicable to 2 groups, and 1 state (Maine) had a mandate applicable to only 1 group.

**Table.** State Vaccine Mandate Announcements and Effective Dates, and Worker Groups Covered by Mandates From July to December 2021

State	Announcement date	Effective date	Worker groups covered^a^				
			Congregate facility	Government	Health care	Long-term care facility	K-12 school workers
California	July 26, 2021	October 14, 2021	X	X	X	X	X
Colorado	July 30, 2021	October 31, 2021	X	X	X	X	
Connecticut	August 6, 2021	October 21, 2021		X	X	X	X
District of Columbia	August 10, 2021	November 1, 2021		X	X		X
Maine	August 12, 2021	September 17, 2021			X		
Massachusetts	August 4, 2021	October 10, 2021		X		X	
Nevada	July 30, 2021	November 1, 2021	X	X			
New Mexico	July 29, 2021	October 20, 2021	X	X	X		X
New York	July 26, 2021	November 10, 2021		X	X	X	X
North Carolina	July 27, 2021	September 30, 2021		X	X		
Oregon	August 5, 2021	October 18, 2021		X	X	X	X
Rhode Island	August 17, 2021	October 1, 2021			X	X	
Washington	August 9, 2021	October 18, 2021		X	X	X	X

^a^ Includes all worker groups covered by any type of vaccine mandate in the state, only for states with at least 1 mandate with no test-out option in effect on or before December 31, 2021.

**Figure 1.**
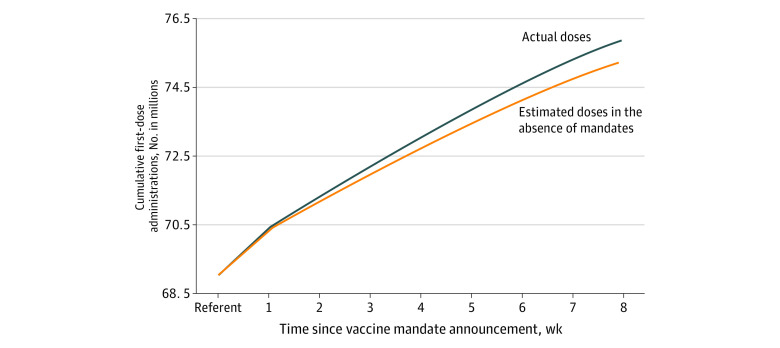
Cumulative First-Dose Administrations by Time Since Vaccine Mandate Announcement Comparing Actual Doses Administered and Estimated Doses in the Absence of Mandates (Counterfactual Doses) in States That Issued Vaccine Mandates (July-October 2021) End-of-week cumulative doses are shown for each period.

At baseline (8 weeks before the announcement), between 47.9% and 69.5% of the population in the mandate group states had received their first dose of COVID-19 vaccine. At the study end point (8 weeks after the announcement), these percentages increased to between 60.4% and 78.6%. In the 8 weeks following a mandate announcement, 5 508 539 people received their first COVID-19 vaccine dose in the mandate group states, of which an estimated 634 831 vaccinations (11.5%) may be attributable to the mandate announcement. The number of people who completed the vaccination series associated with the mandate announcement was not estimated because the percentage point differences were not statistically significant until 7 and 8 weeks after the announcement. The estimated numbers of people in the mandate group states who received their first vaccination dose associated with the mandate announcement are shown in Figure 1 and eTable 1 in the Supplement.

**Figure 2.**
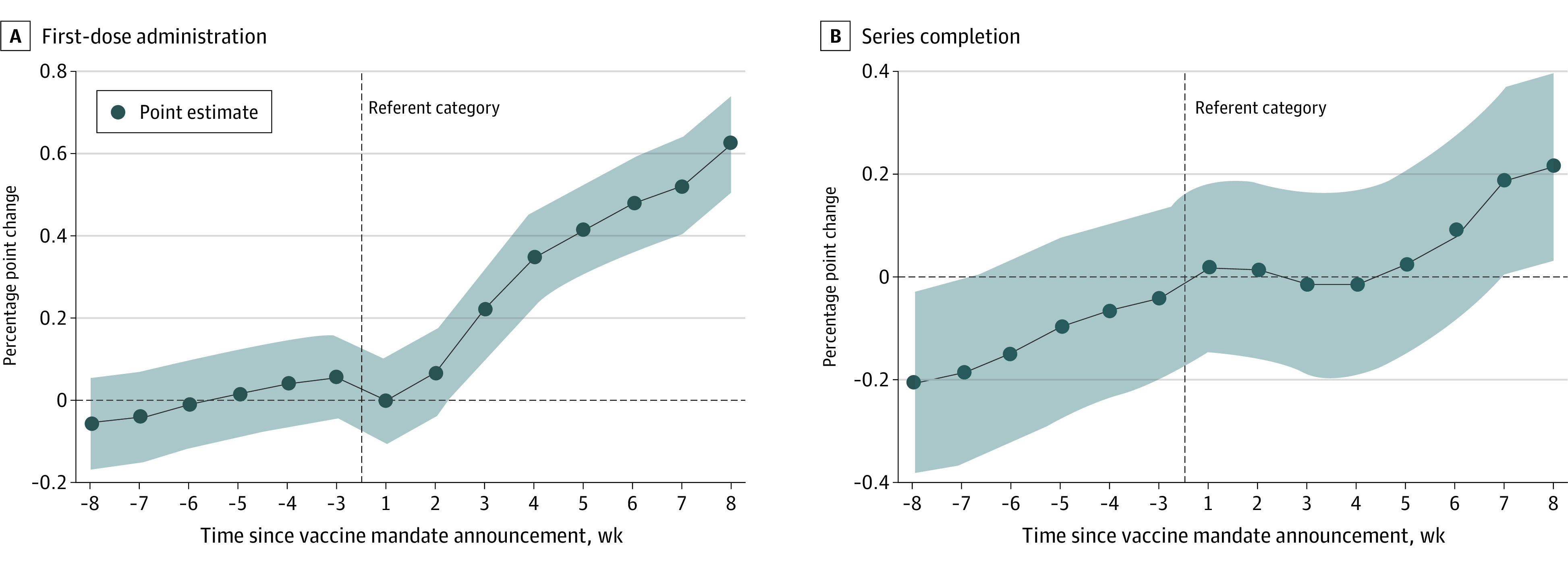
Association Between State-Issued Vaccine Mandates Shown as Adjusted Average Percentage Point Changes of COVID-19 Vaccine First-Dose Administration and Series Completion in 13 States With a Vaccine Mandate Relative to 14 Comparison Group States (May-October 2021) A, State-issued vaccine mandates were defined as requirements for a group of workers to (1) be vaccinated with no test-out option except for those with approved medical or religious exemptions, or (2) be vaccinated and undergo recurring testing (for the states in each group, see the Inclusion Criteria subsection in the Methods section). B, Percentage point changes and 95% CIs estimated from regression models were controlled for state and time (calendar date) fixed effects, previous week rolling 7-day average new cases per 100 000 population, percentage of the population fully vaccinated, and whether the observation occurred before or after the mandate effective date. Percentage point changes are relative to the average growth rates for the referent category (14 comparison group states and the referent period of 0- 2 weeks before the mandate announcement date in states that issued a mandate) denoted by the vertical reference line. For the first-dose administrations model, the model-estimated referent category coverage was 62.9%. For the series completion model, the model-estimated referent category coverage was 56.3%. Shaded areas indicate 95% CI.

Unadjusted weekly averages of daily first-dose administrations and daily vaccine series completion per 100 000 population for the mandate and comparison states are shown in eFigure 1 in the Supplement, while the unadjusted first-dose administration and vaccine series completion coverage for the mandate and comparison group states are shown in eFigure 2 in the Supplement. Results from the regression models are shown as percentage point changes relative to the referent category (the 2 weeks prior to the announcement date in both mandate group and comparison group states) in Figure 2 and eTable 2 in the Supplement. The corresponding 95% CI for the point estimates and a reference line at *y* = 0 are also provided to aid interpretation of the statistical significance of the point estimates. The weekly first-dose administration coverage was similar in the mandate states for all preannouncement periods relative to the referent category. The coverage difference appeared significantly higher than in the referent category beginning 3 weeks after the mandate announcement date. The mandate announcement was associated with an increase in the weekly first-dose administration coverage of 0.20, 0.33, 0.39, 0.45, 0.49, and 0.59 percentage points, 3 to 8 weeks after the announcement (for all comparisons, *P* ≤ .001) relative to the adjusted referent category average coverage of 62.9% (eTable 2 in the Supplement). Vaccine series completion increased in the mandate group states during weeks 3 to 8 after the mandate announcement (Figure 2B; eTable 2 in the Supplement), but these results were statistically different from the referent category only at weeks 7 and 8 after the announcement, when the coverage for both weeks was 0.2 percentage points higher than the adjusted referent category series completion of 56.3% (*P* = .05 and .02, respectively). Scatter plots of observed and fitted values are shown in eFigure 3 in the Supplement.

## Discussion

Our analysis of COVID-19 vaccine mandates and vaccination rates in 13 state-level jurisdictions suggests that these mandates may have been associated with increases in statewide first-dose administration rates. Percentage point differences in the population’s first-dose vaccination coverage were higher than the referent category coverage 3 to 8 weeks following the mandate announcement date, and these differences increased over time. Although coverage steadily increased during the preannouncement period, the steep increase in the weeks immediately following the announcement suggests a direct response to the mandate.

The percentage of the population with vaccine series completion increased 3 to 4 weeks following the increase in first-dose administration rates, consistent with preliminary findings from other countries in which vaccines were mandated for at least some groups.^31^ The rates of change reached statistical significance in weeks 7 and 8. The delayed increase in the growth rates observed for vaccine series completion is consistent with the recommended second-dose timing for primary series completion for 2-dose vaccinations in the periods covered by this study (3-8 weeks between doses for BNT162b2 and 4-8 weeks for mRNA-1273).^26^

The percentage point differences in the series completion coverage started to increase 4 weeks after the mandates were announced; at that time, the CDC recommended 3 to 4 weeks between doses for the 2-dose mRNA vaccine series. Considered with the initial increase in first-dose administration rates, the increase in series completion growth rates is in accordance with the recommended timing of the 2-dose vaccination series.

The mandates in this study were all specific to different groups of workers and did not apply to the general public. In some states, the mandates may have applied to only a single group, such as government employees, while in other states, the mandates applied to multiple groups. The overall increase in vaccination coverage across jurisdictions observed in this study indicates that mandates may have a spillover effect, encouraging others outside of the worker groups directly governed by the mandate to be vaccinated. Although the empirical association between a mandate applicable to any group and a statewide population’s reaction could not be assessed in this study, this finding is consistent with literature suggesting that both mandates and recommendations increase vaccination rates.^7^ We posit that a mandate announced within a state may signal to the population that vaccines are safe and important and may decrease hesitancy. Recipients of first doses that account for the increased growth rates may have done so because: (1) they were already intending to get vaccinated but planned on doing so later, and the mandate encouraged them to seek vaccination immediately; or (2) did not intend to receive the vaccine and did so only in response to the order. The population-level changes observed suggest that an official statement from a government increases short-term vaccination rates, potentially including individuals not directly mandated to be vaccinated.

### Limitations

This study has several limitations. First, more than 1 mandate was issued in many states and may or may not have applied to the same group or groups as the original mandate. These additional mandates may have been introduced at any time from July 26, 2021, to the end of the study period (December 31, 2021) and may have influenced vaccine uptake. The date of the first vaccine mandate announced in the state was used because it is likely to have the greatest impact on behavior, but additional mandates may also have influenced vaccine uptake. This study measured only the presence of a mandate and did not attempt to identify what type or combination of mandates had the largest impact. Second, only states that issued a vaccine mandate that did not allow a test-out option were included in the mandate group. Third, our study was limited to state-issued orders and did not include city or county orders, nor did it consider the efforts to enforce vaccine mandates, such as community and employer outreach efforts. Fourth, this study examined a finite time frame that ended 8 weeks (56 days) after a mandate was announced. The results indicate only the short-term changes in growth rates associated with the mandates and do not suggest any long-term outcomes of the state-issued mandates. Fifth, many of these mandates were announced during the B.1.617.2 (Delta) variant surge; although the models controlled for the previous week’s 7-day rolling average cases per 100 000 population, time (calendar date), and state fixed effects, there may have been other time-varying factors, including motivation for vaccination, that could not be controlled for in the models. Sixth, policy changes may have been endogenous. States that issued a vaccine mandate may already have had high baseline vaccination coverage or vaccine acceptability within the population.

## Conclusions

To our knowledge, this cross-sectional study is the first to analyze the association between state-issued COVID-19 vaccine mandates and rates of vaccination in the US. The results suggest an association between a state’s vaccine mandate announcement and a statewide increase in vaccination coverage, which was observed after controlling for other motivating factors for vaccination, such as increased case incidence during the Delta variant surge, and overall time trends. This observed association may be a product of both a direct outcome for groups governed by the mandate as well as an indirect signal about the importance of vaccination to the general population of the state.

## Notes

Supplement. **eFigure 1**. Unadjusted Average Daily (a) First-Dose Administrations and (b) Vaccine Series Completion for Mandate and Comparison Group States by Calendar Week, May-October 2021 **eFigure 2**. Unadjusted Average Daily (a) First-Dose Administrations and (b) Vaccine Series Completion Coverage for Mandate and Comparison Group States by Calendar Week, May-October 2021 **eTable 1.** Estimated Number of People Receiving First-Dose Vaccination as a Result of State-Issued Vaccine Mandate Announcement, 13 States July-October 2021 **eTable 2.** Association Between State-Issued Vaccine Mandates Shown as Adjusted Average Percentage Point Changes of COVID-19 Vaccine First-Dose Administration and Series Completion in States With a Vaccine Mandate (n = 13) Relative to Control Group States (n = 14), May-October 2021 **eFigure 3.** Observed and Fitted Values of (a) First-Dose Administration and (b) Vaccine Series Completion Coverage
