# Supplementary material for: Association Between State-Issued COVID-19 Vaccine Mandates and Vaccine Administration Rates in 12 US States and the District of Columbia
Source: JAMA Health Forum. 2022 Oct 28;3(10):e223810. doi: 10.1001/jamahealthforum.2022.3810 (PMC9617176; doi:10.1001/jamahealthforum.2022.3810)
Supplement: Supplement. — eFigure 1. Unadjusted Average Daily (a) First-Dose Administrations and (b) Vaccine Series Completion for Mandate and Comparison Group States by Calendar Week, May-October 2021 eFigure 2. Unadjusted Average Daily (a) First-Dose Administrations and (b) Vaccine Series Completion Coverage for Mandate and Comparison Group States by Calendar Week, May-October 2021 eTable 1. Estimated Number of People Receiving First-Dose Vaccination as a Result of State-Issued Vaccine Mandate Announcement, 13 States July-October 2021 eTable 2. Association Between State-Issued Vaccine Mandates Shown as Adjusted Average Percentage Point Changes of COVID-19 Vaccine First-Dose Administration and Series Completion in States With a Vaccine Mandate (n = 13) Relative to Control Group States (n = 14), May-October 2021 eFigure 3. Observed and Fitted Values of (a) First-Dose Administration and (b) Vaccine Series Completion Coverage [file jamahealthforum-e223810-s001.pdf]

Supplementary Online Content

Howard-Williams M, Soelaeman RH, Fischer LS, McCord R, Davison R, Dunphy C. Association between state-issued COVID-19 vaccine mandates and vaccine administration rates in 12 US states and the District of Columbia. *JAMA Health Forum.* 2022;3(10): e223810. doi:10.1001/jamahealthforum.2022.3810

eFigure 1. Unadjusted Average Daily (a) First-Dose Administrations and (b) Vaccine Series Completion for Mandate and Comparison Group States by Calendar Week, May-October 2021

eFigure 2. Unadjusted Average Daily (a) First-Dose Administrations and (b) Vaccine Series Completion Coverage for Mandate and Comparison Group States by Calendar Week, May-October 2021

eTable 1. Estimated Number of People Receiving First-Dose Vaccination as a Result of State-Issued Vaccine Mandate Announcement, 13 States July-October 2021

eTable 2. Association Between State-Issued Vaccine Mandates Shown as Adjusted Average Percentage Point Changes of COVID-19 Vaccine First-Dose Administration and Series Completion in States With a Vaccine Mandate (n = 13) Relative to Control Group States (n = 14), May-October 2021

eFigure 3. Observed and Fitted Values of (a) First-Dose Administration and (b) Vaccine Series Completion Growth Rates

This supplementary material has been provided by the authors to give readers additional information about their work.

© 2022 Howard-Williams M et al. *JAMA Health Forum.*

eFigure 1. Unadjusted Average Daily (a) First-Dose Administrations and (b) Vaccine Series Completion for Mandate and Comparison Group States by Calendar Week, May-October 2021

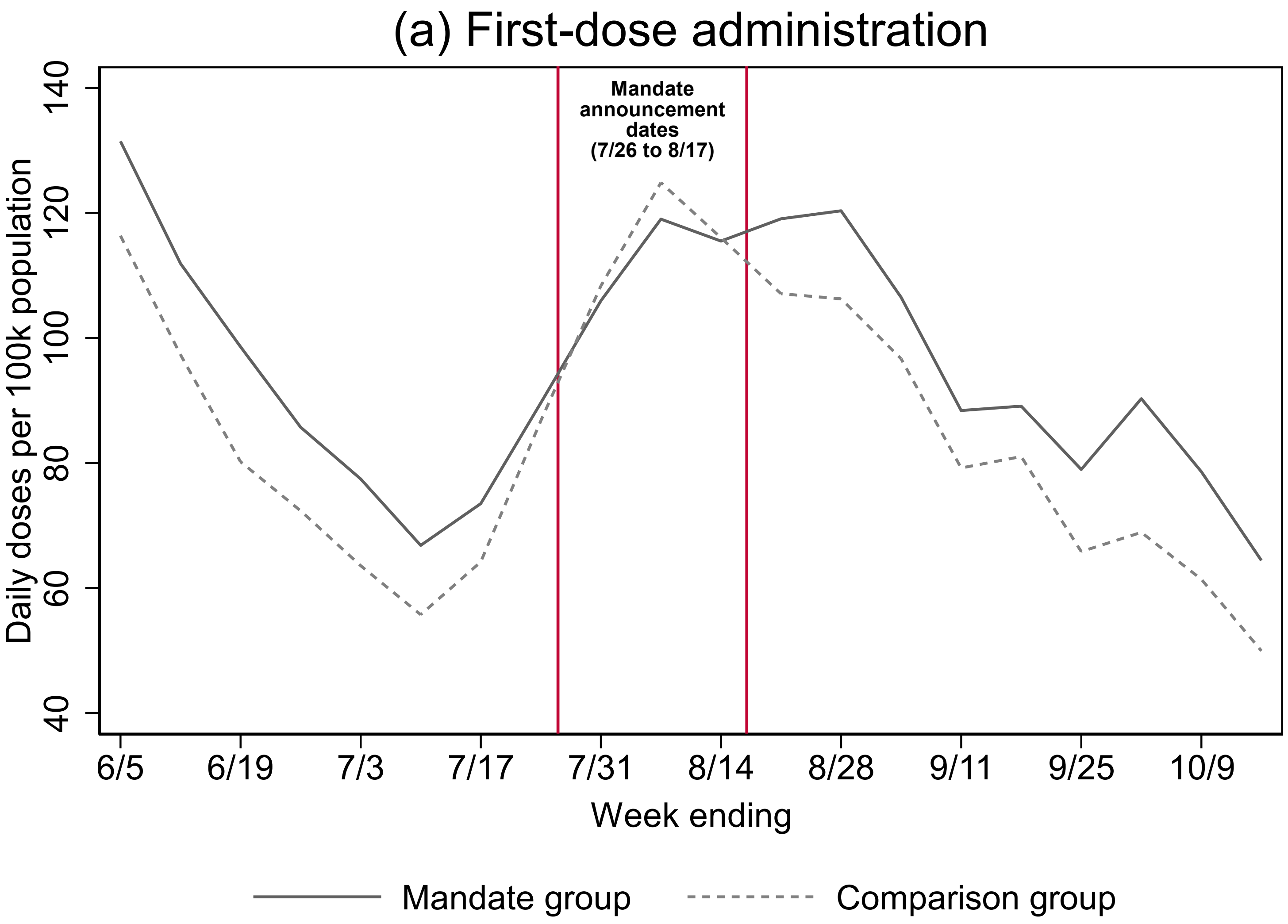

© 2022 Howard-Williams M et al. *JAMA Health Forum.*

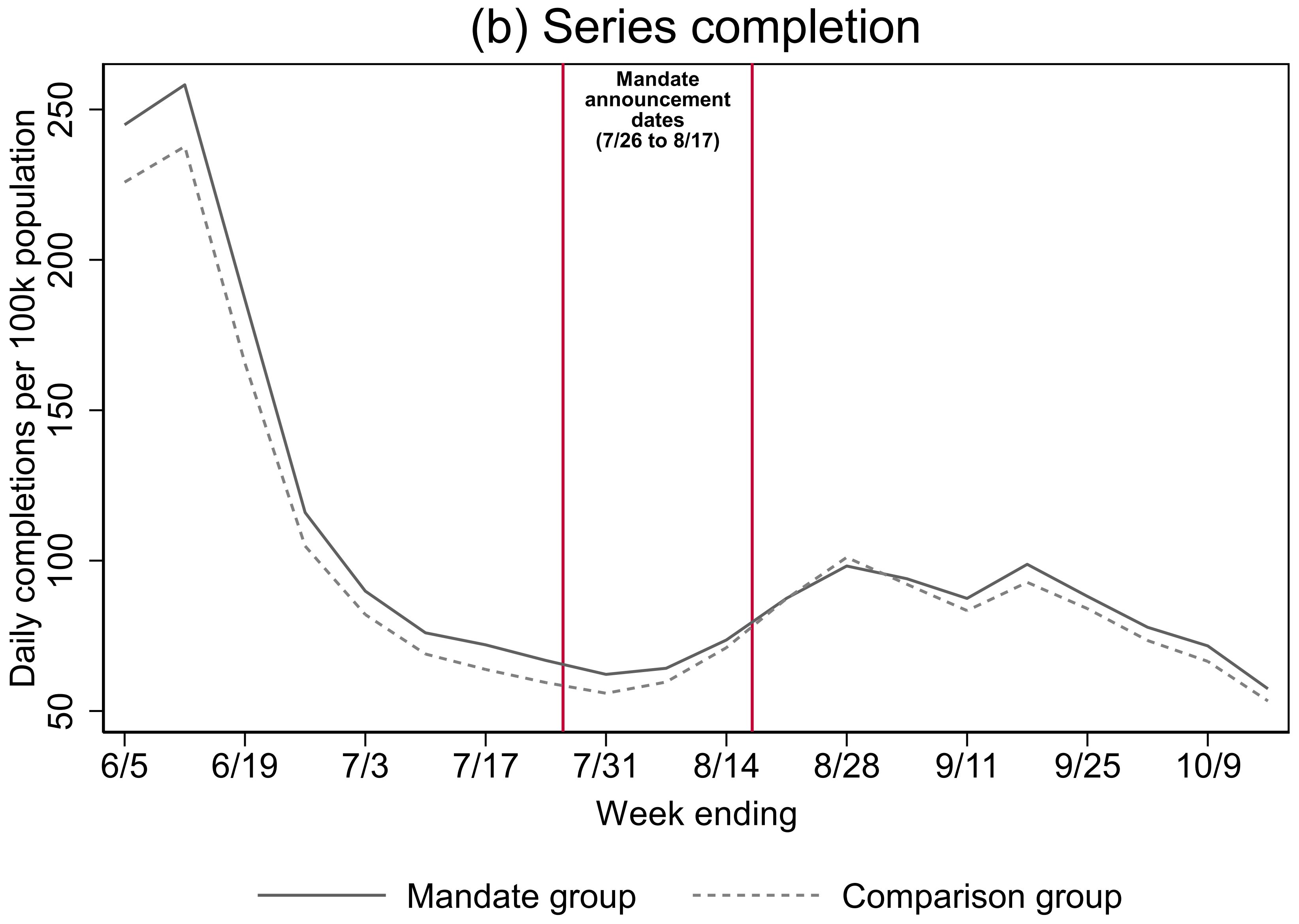

© 2022 Howard-Williams M et al. *JAMA Health Forum.*

eFigure 2. Unadjusted Average Daily (a) First-Dose Administrations and (b) Vaccine Series Completion Coverage for Mandate and Comparison Group States by Calendar Week, May-October 2021

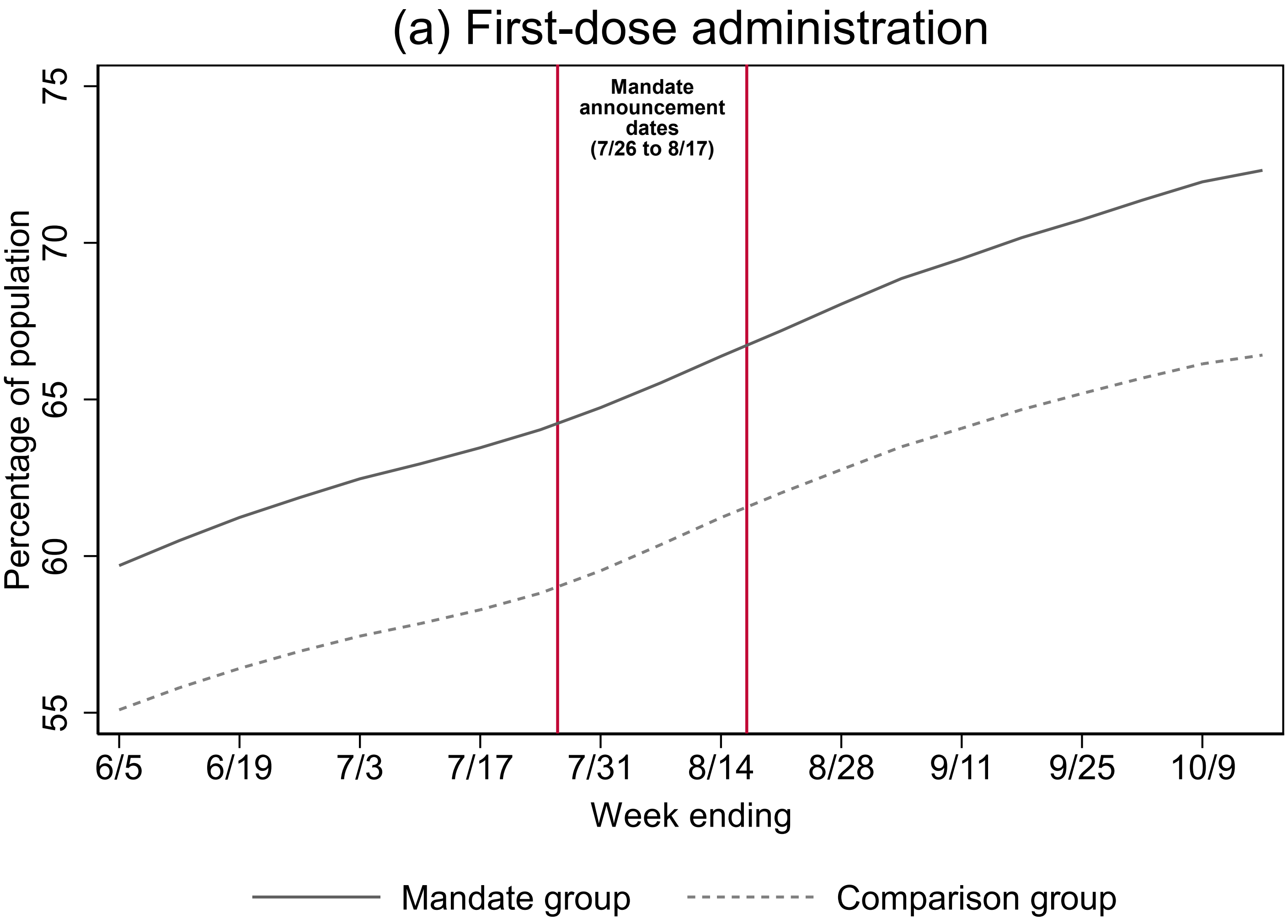

© 2022 Howard-Williams M et al. *JAMA Health Forum.*

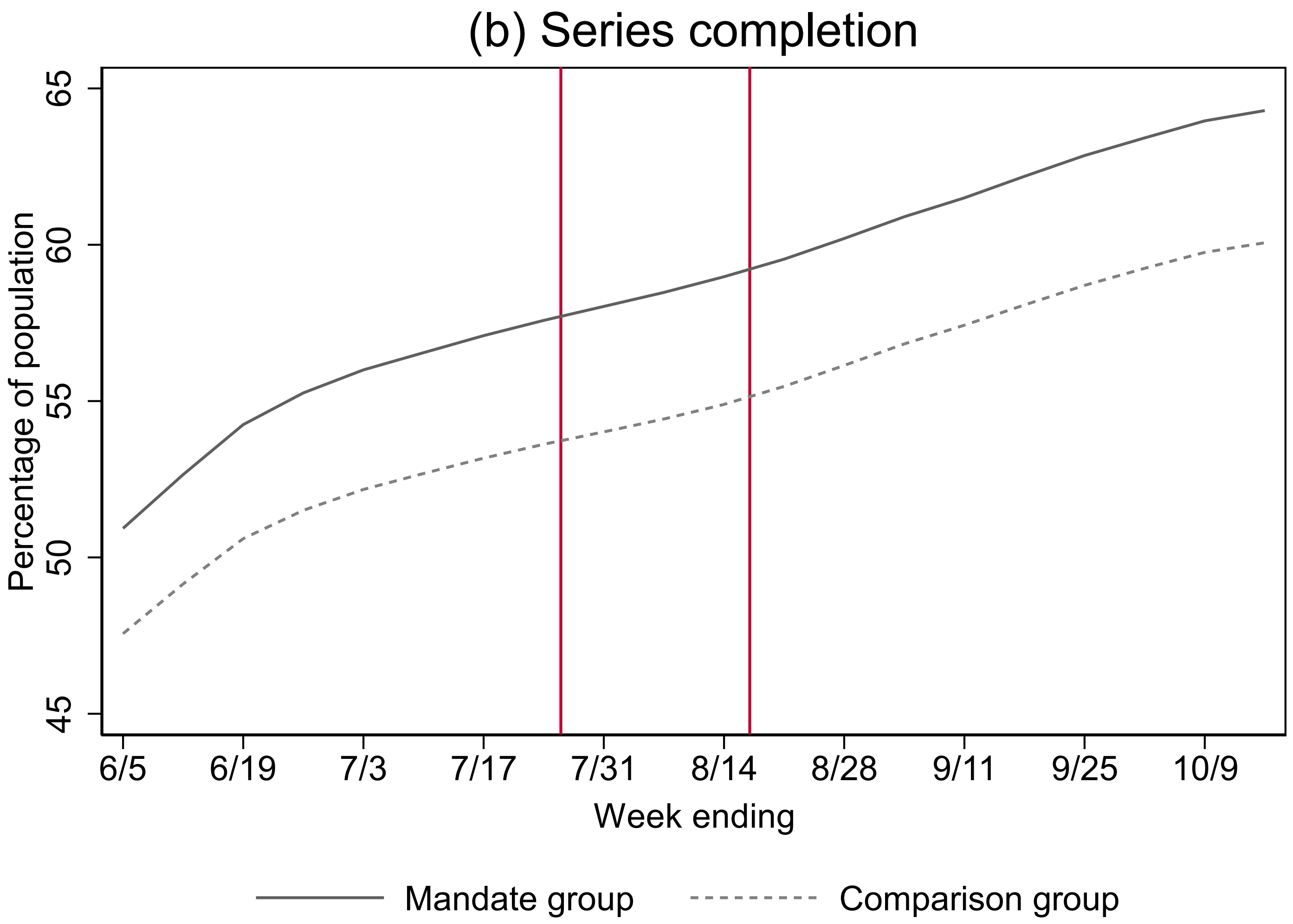

© 2022 Howard-Williams M et al. *JAMA Health Forum.*

eTable 1. Estimated Number of People Receiving First-Dose Vaccination as a Result of State-Issued Vaccine Mandate[a] Announcement, 13 States July-October 2021

Time since vaccine mandate announcement	Actual – Mandate Announced			Counterfactual – No Mandate Announced[c]			Cumulative mandate-associated first-dose administrations at end of week[d]
	Percentage of population covered at end of week	Cumulative number of people covered at end of week[b]		Percentage of population covered at end of week	Cumulative number of people covered at end of week[b]		
1 week	65.9	70,383,809		65.9	70,393,305		-9,497
2 weeks	66.7	71,292,699		66.7	71,231,475		61,225
3 weeks	67.6	72,208,643		67.4	71,990,691		217,952
4 weeks	68.4	73,079,952		68.1	72,729,291		350,660
5 weeks	69.1	73,884,334		68.8	73,468,887		415,448
6 weeks	69.8	74,592,426		69.4	74,108,020		484,406
7 weeks	70.4	75,252,367		69.9	74,724,541		527,827
8 weeks	71.0	75,892,347		70.4	75,257,516		634,831

a. State-issued vaccine mandates were defined as requirements for a group of workers to 1) be vaccinated with no test-out option except for those with approved medical or religious exemptions, or 2) be vaccinated and undergo recurring testing. States with at least one mandate that did not allow a test-out option in effect prior to December 31, 2021 were categorized as the mandate group. They were California, Colorado, Connecticut, the District of Columbia, Maine, Massachusetts, Nevada, New Mexico, New York, North Carolina, Oregon, Rhode Island, Washington. States included in the comparison group either a) issued a mandate allowed a test-out option or b) did not issue any type of mandate and did not otherwise restrict mandates such as by issuing mandate prohibitions. They were Delaware, Hawaii, Illinois, Kentucky, Louisiana, Maryland, Minnesota, New Jersey, Ohio, Pennsylvania, Virginia, Vermont, West Virginia, and Wisconsin.

b. The cumulative number of people covered at the end of the week was computed by taking the percentage of population covered at the end of the week with the combined population of the 13 mandate group states (n= 106,859,204).

c. The counterfactual percentage of population covered at the end of the week was computed by subtracting the regression model coefficients presented in eTable 2 from the actual/observed percentage of population covered at the end of the week.

d. The cumulative mandate-associated first-dose administrations at the end of the week was computed by taking the difference between the actual and counterfactual numbers of people covered at the end of the week.

© 2022 Howard-Williams M et al. *JAMA Health Forum.*

eTable 2. Association Between State-Issued Vaccine Mandates[a] Shown as Adjusted Average Percentage Point Changes of COVID-19 Vaccine First-Dose Administration and Series Completion[b] in States with a Vaccine Mandate (n = 13) Relative to Control Group States (n = 14), May-October 2021

Time from mandate announcement	First-dose administration			Series completion	
	**Percentage point change (95% confidence interval)**	**p-value**		**Percentage point change (95% confidence interval)**	**p-value**
8 weeks before	-0.06 (-0.17 to 0.05)	0.26		-0.21 (0.09 to -0.03)	0.02
7 weeks before	-0.05 (-0.15 to 0.06)	0.39		-0.19 (-0.36 to -0.01)	0.04
6 weeks before	-0.02 (-0.12 to 0.09)	0.76		-0.15 (-0.32 to 0.03)	0.10
5 weeks before	0.01 (-0.10 to 0.11)	0.91		-0.10 (-0.27 to 0.08)	0.27
4 weeks before	0.03 (-0.07 to 0.13)	0.54		-0.07 (-0.23 to 0.10)	0.45
3 weeks before	0.05 (-0.05 to 0.15)	0.34		-0.04 (0.08 to 0.12)	0.63
0 to 2 weeks before in intervention states + control group states	Referent			Referent	
1 week after	-0.01 (-0.11 to 0.09)	0.86		0.02 (-0.15 to 0.18)	0.82
2 weeks after	0.06 (-0.05 to 0.16)	0.28		0.01 (-0.16 to 0.18)	0.88
3 weeks after	0.20 (0.10 to 0.31)	<0.001		-0.01 (-0.19 to 0.16)	0.88
4 weeks after	0.33 (0.22 to 0.43)	<0.001		-0.01 (-0.19 to 0.16)	0.87
5 weeks after	0.39 (0.28 to 0.50)	<0.001		0.02 (-0.15 to 0.20)	0.80
6 weeks after	0.45 (0.34 to 0.56)	<0.001		0.09 (-0.09 to 0.27)	0.31
7 weeks after	0.49 (0.38 to 0.60)	<0.001		0.19 (0.004 to 0.37)	0.05
8 weeks after	0.59 (0.48 to 0.70)	<0.001		0.21 (0.03 to 0.40)	0.02
Number of state-day observations used	*3,346*			*3,346*	

a. State-issued vaccine mandates were defined as requirements for a group of workers to 1) be vaccinated with no test-out option except for those with approved medical or religious exemptions, or 2) be vaccinated and undergo recurring testing. States with at least one mandate that did not allow a test-out option in effect prior to December 31, 2021 were categorized as the intervention group. They were California, Colorado, Connecticut, the District of Columbia, Maine, Massachusetts, Nevada, New Mexico, New York, North Carolina, Oregon, Rhode Island, Washington. States included in the control group either a) issued a mandate allowed a test-out option or b) did not issue any type of mandate and did not otherwise restrict mandates such as by issuing prohibitions. They were Delaware, Hawaii, Illinois, Kentucky, Louisiana, Maryland, Minnesota, New Jersey, Ohio, Pennsylvania, Virginia, Vermont, West Virginia, and Wisconsin.

b. Percentage point changes are coefficients from regression models controlling for state and time (calendar date) fixed effects, previous week rolling 7-day average new cases per 100,000 population, percentage of population fully vaccinated, and whether the observation occurred before or after mandate effective date. Percentage point changes are relative to the coverage for the **referent category** (14 control group states and the referent period of 0 to 2 weeks before mandate announcement date in states that issued a mandate) denoted by the vertical reference line. For the first-dose administrations model, the model-estimated referent category coverage62.9%. For the series completion model, the model-estimated referent category coverage was 56.3%.

© 2022 Howard-Williams M et al. *JAMA Health Forum.*

eFigure 3. Observed and Fitted Values of (a) First-Dose Administration and (b) Vaccine Series Completion Coverage

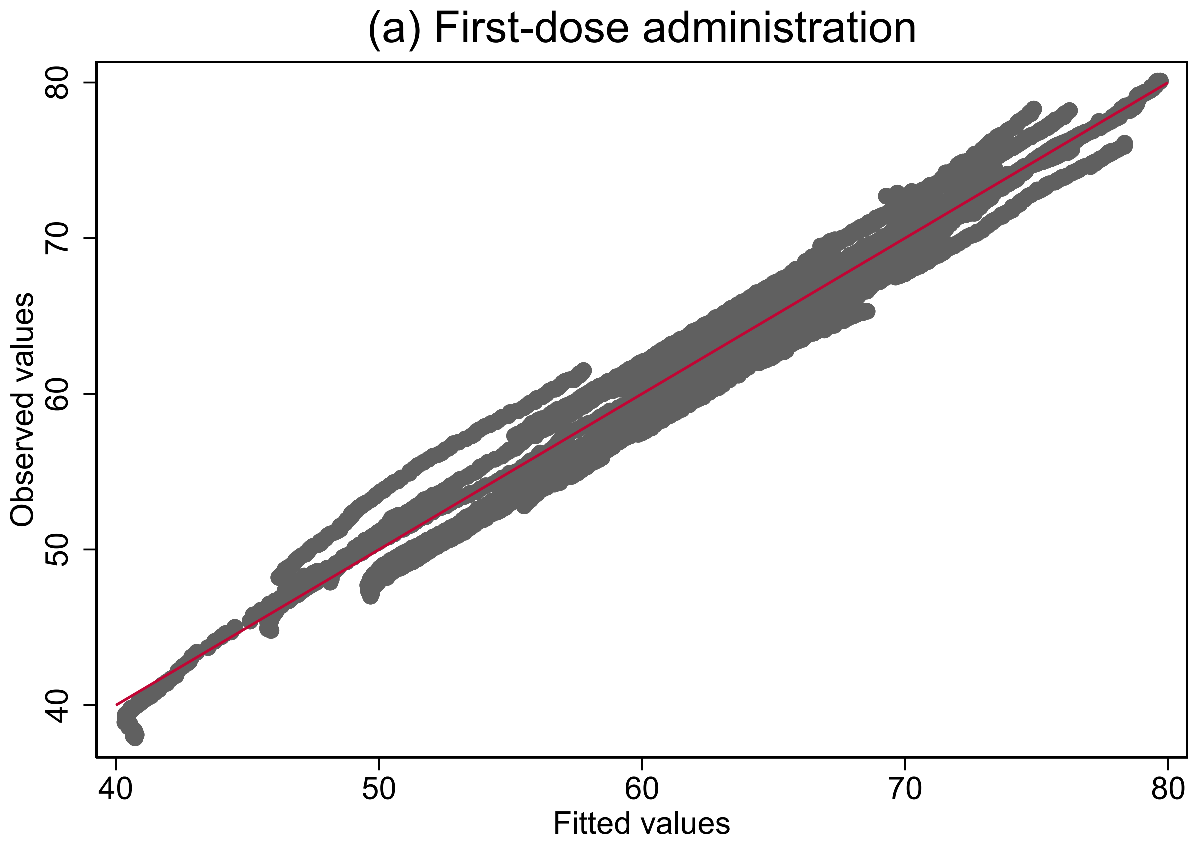

© 2022 Howard-Williams M et al. *JAMA Health Forum.*

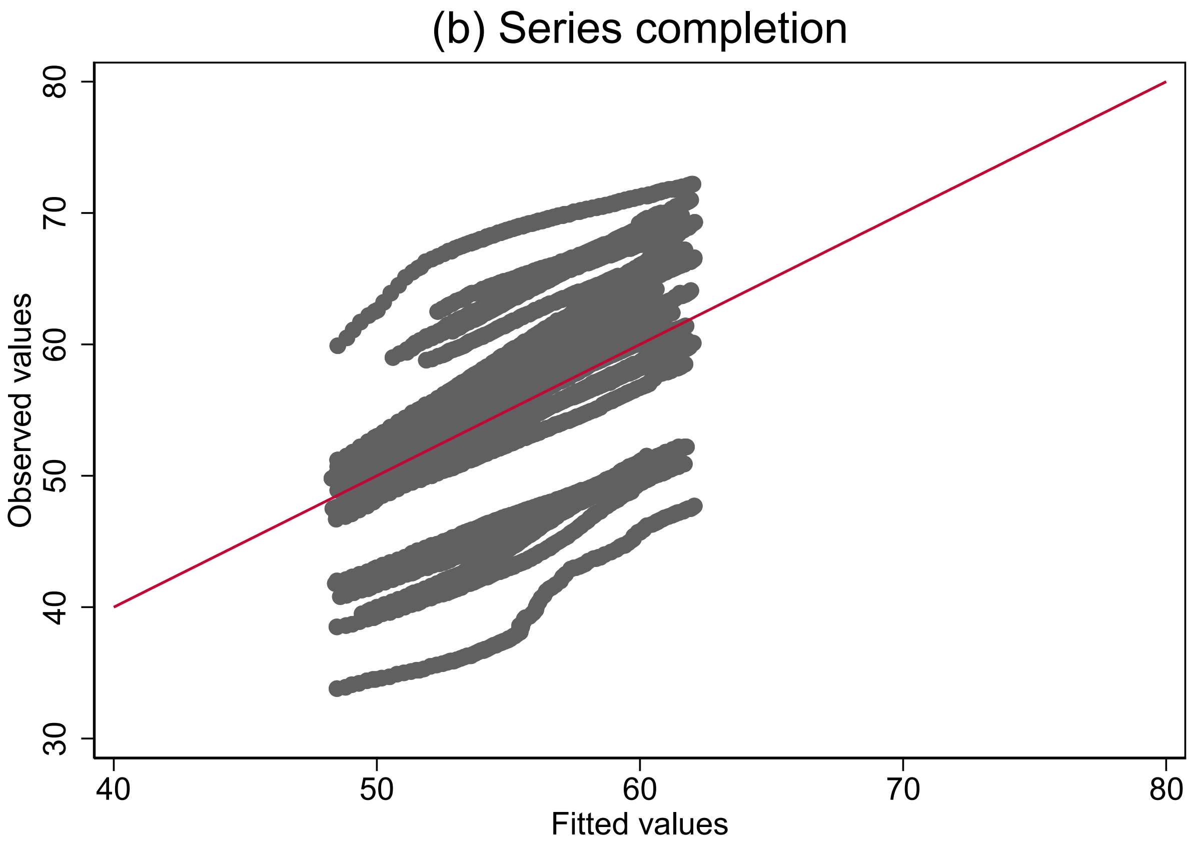

© 2022 Howard-Williams M et al. *JAMA Health Forum.*
